# Factors Associated with Antimicrobial Drug Use in Medicaid Programs

**DOI:** 10.3201/eid2005.130493

**Published:** 2014-05

**Authors:** Pengxiang Li, Joshua P. Metlay, Steven C. Marcus, Jalpa A. Doshi

**Affiliations:** University of Pennsylvania, Philadelphia, Pennsylvania, USA

**Keywords:** Medicaid, acute respiratory tract infections, factors, antimicrobial drug use

## Abstract

Using US Medicaid data, we found that 52% of adult Medicaid patients with acute respiratory tract infections filled prescriptions for antimicrobial drugs in 2007. Factors associated with lower likelihood of use were higher county-level availability of primary care physicians and state-level participation in a campaign for appropriate antimicrobial drug use.

Antimicrobial drugs are not recommended for the treatment of acute respiratory tract infections (ARIs), such as colds, upper respiratory tract infections (URIs), and acute bronchitis (*1*,*2*). Unnecessary use contributes to emergence of antimicrobial drug–resistant bacteria (*1*), an emerging public health crisis (*2*) that contributes to greater rates of illness and death and economic costs as high as $4 billion/year (*3*).

Inappropriate use of antimicrobial drugs in Medicaid programs is a potentially serious problem (*4*,*5*). Medicaid is a US health insurance program that covers 58 million low-income persons and families (*6*). The number of enrolled adults is expected to increase substantially as a result of the Patient Protection and Affordable Care Act (*7*). In this study, we estimated the rate and factors associated with antimicrobial drug use for the treatment of ARIs among adult Medicaid enrollees.

## The Study

We used the 2007 Medicaid Analytic Extract files for patients >21 years of age from 40 states linked with the Area Resource File. Index visits were identified as the first visit to a physician during the study period when a primary diagnoses of ARI was made (cold, acute URIs at multiple unspecified sites, or acute bronchitis) (*8*,*9*). The identification period for the index visit was January 1, 2007, through December 24, 2007. We excluded patients who received index visit diagnoses for which antimicrobial drugs were appropriate (Technical Appendix Figure 1).

The outcome variable was presence or absence of a claim for an antimicrobial drug prescription linked to the index visit. Drug classes considered were cephalosporins, penicillins, sulfonamides, macrolides (including azalides), lincosamides, tetracyclines, and quinolones (*4*). Similar to most prescription claims data, Medicaid drug claims do not list a diagnosis that corresponds to the indication for treatment. Hence, the drug was presumed to have been prescribed for an ARI if the prescription was filled on the same date that the patient visited the physician for the ARI or within 4 days of this index visit (*8*).

Logistic regression analyses with robust estimation, adjusting for state-level clustering, were used to identify factors associated with antimicrobial drug prescriptions for ARI visits. Covariates included patient age, sex, race, and the Prescription Drug Hierarchical Coexisting Condition score as a measure of concurrent conditions and medication need (*10*) and county-level covariates. To account for seasonal effects, we included indicators for the quarter in which the index visit occurred. The density of primary care physicians in the county of the beneficiary’s residence was measured from the Area Resource File as the number of general practice, family medicine, and general internal medicine physicians per 10,000 persons. This measure was coded as categorical variables according to the quintile of the measure across all counties in the Area Resource File. An indicator variable identified whether the patient resided in a state that was funded by the Centers for Disease Control and Prevention (CDC) Get Smart: Know When Antibiotics Work campaign for appropriate antimicrobial drug use within the 5 years before the study (*11*). Sensitivity analyses varied the covariates included in the regression analyses and the time window (3–7 days after index visit) for linking the drugs to the URI diagnosis (*5*,*9*). Subgroup analyses were conducted among patients <65years of age and patients without diabetes or congestive heart failure.

In 2007, a total of 194,874 adults had at least 1 physician visit at which a primary diagnosis of ARI was made with no other associated secondary diagnoses for which treatment with an antimicrobial drug would be appropriate (Table 1). After this visit, ≈52% of patients filled an antimicrobial drug prescription (Figure). The most common prescriptions filled were for macrolides (27.8%) and penicillins (12.3%).

**Table 1:** Characteristics of 194,874 adult Medicaid patients with acute respiratory tract infection, 40 US states, January 1–December 24, 2007*

Variable	No. (%) or mean (SD)
Age, y, no. (%)	
21–29	45,447 (23.3)
30–39	42,977 (22.1)
40–49	42,924 (22)
50–59	38,376 (19.7)
60–64	16,929 (8.7)
>65	8,221 (4.2)
Sex, no. ( %)	
F	143,329 (73.5)
M	51,545 (26.5)
Race, no. ( %)	
White	100,310 (51.5)
Black	46,282 (23.7)
Hispanic	16,404 (8.4)
Other	31,878 (16.4)
Diagnosis at index visit, no. (%)	
Cold or acute URIs (ICD-9 codes 460 and 465)	113,394 (58.2)
Acute bronchitis (ICD-9 code 466)	81,480 (41.8)
RxHCC score, mean (SD)†	0.5 (0.6)
Quarter of index visit date, no. (%)	
Jan–Mar	85,601 (43.9)
Apr–Jun	36,771 (18.9)
Jul–Sep	30,807 (15.8)
Oct–Dec	41,695 (21.4)
Residence in low-education county, no. (%)‡	
No	144,335 (74.1)
Yes	50,539 (25.9)
County-level annual per capita income, mean (SD)	32,700 (15.6)
Residence in urban area, no. (%)	
No	65,766 (33.7)
Yes	129,108 (66.3)
Residence in state participating in CDC Get Smart campaign, no. (%)§	
No	38,332 (19.7)
Yes	156,542 (80.3)
Primary care physicians/10,000 persons in county, no. (%)¶	
<2.2	14,816 (7.6)
2.2–3.4	28,500 (14.6)
3.5–4.7	34,017 (17.5)
4.8–6.5	47,460 (24.4)
>6.5	70,081 (36)

*Data from the 2007 Medicaid Analytic Extract files linked with the Area Resource File. URI, upper respiratory tract infection; ICD-9, International Classification of Diseases, Ninth Revision; RxHCC, prescription drug Hierarchical Coexisting Condition; CDC, Centers for Disease Control and Prevention. †Modified RxHCC score used here, wherein coefficients for age and sex are zeroed out in the score calculation because regression models separately control for these variables. Range in sample described here 0–5.3. A higher score indicates a higher medical comorbidity burden. ‡County with >25% adults without a high school diploma. §In this sample, 33 of 40 states participated in the CDC Get Smart campaign during 2002–2006. ¶Categories were based on quintile of county-level number of primary care physicians/10,000 persons. Each category includes 644 counties.

**Figure:**
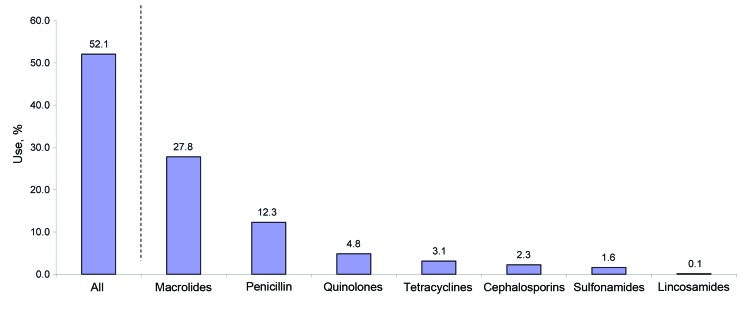
Percentage of antimicrobial drug use, by type of agent, among 194,874 adult Medicaid patients in 40 US state Medicaid programs. Data are from the 2007 Medicaid Analytic Extract files.

Odds of filling antimicrobial drug prescriptions for treatment of ARI were significantly lower for older adults, men, and nonwhite patients (Table 2). Patients with acute bronchitis were substantially more likely than patients with a cold or URI to fill these prescriptions (69% vs. 40%; odds ratio [OR] 3.32; 95% CI 2.78–3.95). Odds of filling antimicrobial drug prescriptions were significantly lower for patients residing in a county for which the quintile for primary care physician density was highest than for patients in a county for which the quintile was lowest (48.2% vs. 56.8%; OR 0.76, 95% CI 0.66–0.88). Likelihood of filling antimicrobial drug prescriptions was lower for patients in 33 states that had participated in the CDC Get Smart campaign during 2002–2006 than for those in other states (50.7% vs. 57.7%; OR 0.74, 95% CI 0.62–0.88). Results of all sensitivity and subgroup analyses were consistent with main results (Technical Appendix Table 1).

**Table 2:** Factors associated with antimicrobial drug use among 194,874 adult Medicaid patients, 40 US states, 2007*

Variable	% Visits at which drugs were prescribed	Odds ratio (95% CI)
Age, y		
21–29	49.8	Referent
30–39	53.7	1.10 (1.05–1.16)
40–49	53.8	1.09 (0.99–1.20)
50–59	53.3	1.09 (0.96–1.23)
60–64	51.3	1.02 (0.89–1.16)
≥65	43.2	0.77 (0.63–0.94)
Sex		
F	52.4	Referent
M	51.1	0.94 (0.90–0.99)
Race		
White	56.9	Referent
Black	48.9	0.82 (0.74–0.91)
Hispanic	45.6	0.73 (0.64–0.82)
Other	45.0	0.75 (0.61–0.92)
Diagnosis at index visit		
Cold or acute URI, ICD-9 codes 460 and 465	40.0	Referent
Acute bronchitis, ICD-9 code 466	69.0	3.32 (2.78–3.95)
RxHCC score†		0.94 (0.88–1.00)
Quarter of index visit date		
Jul–Sep	52.8	Referent
Jan–Mar	52.3	1.04 (1.01–1.08)
Apr–Jun	52.1	1 (0.97–1.04)
Oct–Dec	51.1	0.98 (0.95–1.01)
Residence in low-education county‡		
No	52.1	Referent
Yes	52.1	0.96 (0.85–1.09)
County–level annual per capita income (in $1,000)§		1.00 (1.00–1.00)
Residence in urban area		
No	55.8	Referent
Yes	50.2	0.91 (0.82–1.00)
Residence in state participating in CDC Get Smart campaign¶		
No	57.7	Referent
Yes	50.7	0.74 (0.62–0.88)
No. primary care physicians/10,000 persons in county#		
<2.2	56.8	Referent
2.2–3.4	56.2	0.96 (0.87–1.07)
3.5–4.7	55.4	0.91 (0.80–1.04)
4.8–6.5	51.4	0.84 (0.73–0.96)
>6.5	48.2	0.76 (0.66–0.88)

*Data are from the 2007 Medicaid Analytic Extract files linked with the Area Resource File. URI, upper respiratory tract infection; ICD-9, International Classification of Diseases, Ninth Revision; RxHCC, prescription drug Hierarchical Coexisting Condition; CDC, Centers for Disease Control and Prevention. †Modified RxHCC score used here, wherein coefficients for age and sex are zeroed out in the score calculation because regression models separately control for these variables. Range in sample described here 0–5.3. A higher score indicates a higher medical comorbidity burden.  Odds ratio indicates the increased odds of antimicrobial drug use associated with per unit increase in the score. ‡The categories were based on quintile of county-level number of PCP physicians per 10,000 persons. Each category includes 644 counties. §Defined as a county with ≥25% adults without a high school diploma. ¶In separate analysis, the county-level annual per capita income was coded as categorical variables according to the quintile of the measure across all counties in the Area Resource File. Similar to the continuous variables, the categorical variables were not significant. #In our sample, 33 of 40 states participated in the CDC Get Smart campaign during 2002–2006.

## Conclusion

In 2007, more than half of Medicaid patients filled a prescription for antimicrobial drugs for an ARI, despite essentially no evidence of efficacy for this use. The substantially higher use of antimicrobial drugs for acute bronchitis than for colds or other URIs raises the need for effective interventions to further support physician decision making. Examples of such interventions include active clinician education strategies (e.g., academic detailing, educational workshops, and consensus-building sessions), which are more effective than passive education strategies (e.g., distribution of educational materials) (*1*).

Lower availability of primary care physicians might be associated with higher rates of antimicrobial drug prescribing for ARIs, given that clinicians in areas with fewer primary care physicians see more patients. Physicians with greater patient workloads might be more likely to prescribe antimicrobial drugs for ARIs (*1*), given that they do not have time to counsel patients against use of antimicrobial drugs. This finding has implications for Medicaid because enrollment is expected to increase substantially by 2019 under the Patient Protection and Affordable Care Act. Whether the number of primary care physicians will be adequate to meet this increased demand is a serious concern. In addition, a recent survey showed that nearly one third of physicians are unwilling to see new Medicaid patients (*12*). As a result, inappropriate antimicrobial drug use in Medicaid programs might increase, especially where primary care physician density is low.

Use of antimicrobial drugs for ARIs was lower among patients in states that participated in the CDC Get Smart campaign during 2002–2006. Under this program, CDC helped fund development and implementation of local campaigns to promote appropriate use of antimicrobial drugs. Audiences included providers and patients (*11*). Adding patient education to an existing physician-centered intervention reduces antimicrobial drug use among adults with acute bronchitis (*13*). This finding suggests that such public health campaigns might be associated with lower unnecessary antimicrobial drug use.

Among study limitations are use of administrative claims data, which are collected for purposes of payment rather than research; thus, coding of URI diagnoses might be questionable. Nevertheless, numerous claims-based studies have identified URIs by using International Classification of Diseases, Ninth Revision codes in claims data (*8*,*9*), and a validation study that used chart review showed that for URIs, specificity for these codes was >0.97 (95% CI 0.95–0.98) and sensitivity was 0.56 (95% CI 0.45–0.67) (*14*). Given the cross-sectional study design, our findings associated with primary care physician density and the CDC Get Smart campaign cannot be considered causal. Furthermore, our use of prescription-fill data as a proxy for medication use might have overestimated usage rates. However, this approach has been validated and widely used in studies of medication use (*15*).

That a high percentage of adult Medicaid enrollees with ARIs received antimicrobial drugs unnecessarily raises concern about further widespread use with the upcoming expansion in Medicaid enrollment under health care reform. Clinicians, public health officials, and policymakers should consider ways to curb inappropriate antimicrobial drug use in this population.

Technical Appendix Sample selection diagram and sensitivity and subgroup analyses (odds ratios for primary care physician density and Centers for Disease Control and Prevention Get Smart campaign).
