# Supplementary material for: Factors Associated with Antimicrobial Drug Use in Medicaid Programs
Source: Emerg Infect Dis. 2014 May;20(5):829–32. doi: 10.3201/eid2005.130493 (PMC4012788; doi:10.3201/eid2005.130493)
Supplement: Technical Appendix — Sample selection diagram and sensitivity and subgroup analyses (odds ratios for primary care physician density and Centers for Disease Control and Prevention Get Smart campaign). [file 13-0493-Techapp-s1.pdf]

Article DOI: http://dx.doi.org/10.3201/eid2005.130493

Unnecessary Use of Antimicrobial Drugs by Medicaid Programs

Technical Appendix

Technical Appendix Figure (next page). Sample selection diagram. Notes: a: Index visits were identified as the first office visit during the study period with a primary diagnosis of cold (International Classification of Diseases, Ninth Revision, Clinical Modification [ICD-9-CM], code 460); acute URIs of multiple, unspecified sites (ICD-9-CM code 465); or acute bronchitis (ICD-9-CM code 466), which we collectively refer to as ARIs *(1-9)*. The identification period for the index visit was January 1, 2007, to December 24, 2007. b: Any visits with the relevant primary diagnosis code that occurred within 30 days of a prior visit for any acute respiratory infections (ICD-9-CM codes 460–466) were excluded.*(5)*; Any visits with antibiotic-appropriate secondary diagnoses were also excluded. These codes included suppurative otitis media (ICD-9-CM code 382), nonsuppurative otitis media (ICD-9-CM code 381–381.4), chronic sinusitis (ICD-9-CM code 473), acute sinusitis (ICD-9-CM code 461), acute tonsillitis (ICD-9-CM code 463), acute pharyngitis (ICD-9-CM code 462), pneumonia (ICD-9-CM codes 481–486), streptococcal sore throat (ICD-9-CM code 034.0), urinary tract infections (ICD-9-CM codes 590, 595, 597, and 599.0), bacterial infections (ICD-9-CM code 041), emphysema (ICD-9-CM code 492), and chronic bronchitis (ICD-9-CM code 491).*(10)* Patients with diagnosis claims for chronic obstructive pulmonary disease (ICD-9-CM codes 491.20–491.21, 492.0–492.8, 494, 495.0–495.9, and 496) or asthma (ÍCD-9-CM code 493) within 1 year before and 7 days after the index visit were also excluded.*(1)* Finally, we also excluded index visits that were followed within 7 days by a visit for any of the listed antibiotic-appropriate diagnoses to further avoid misclassification of simple ARIs.

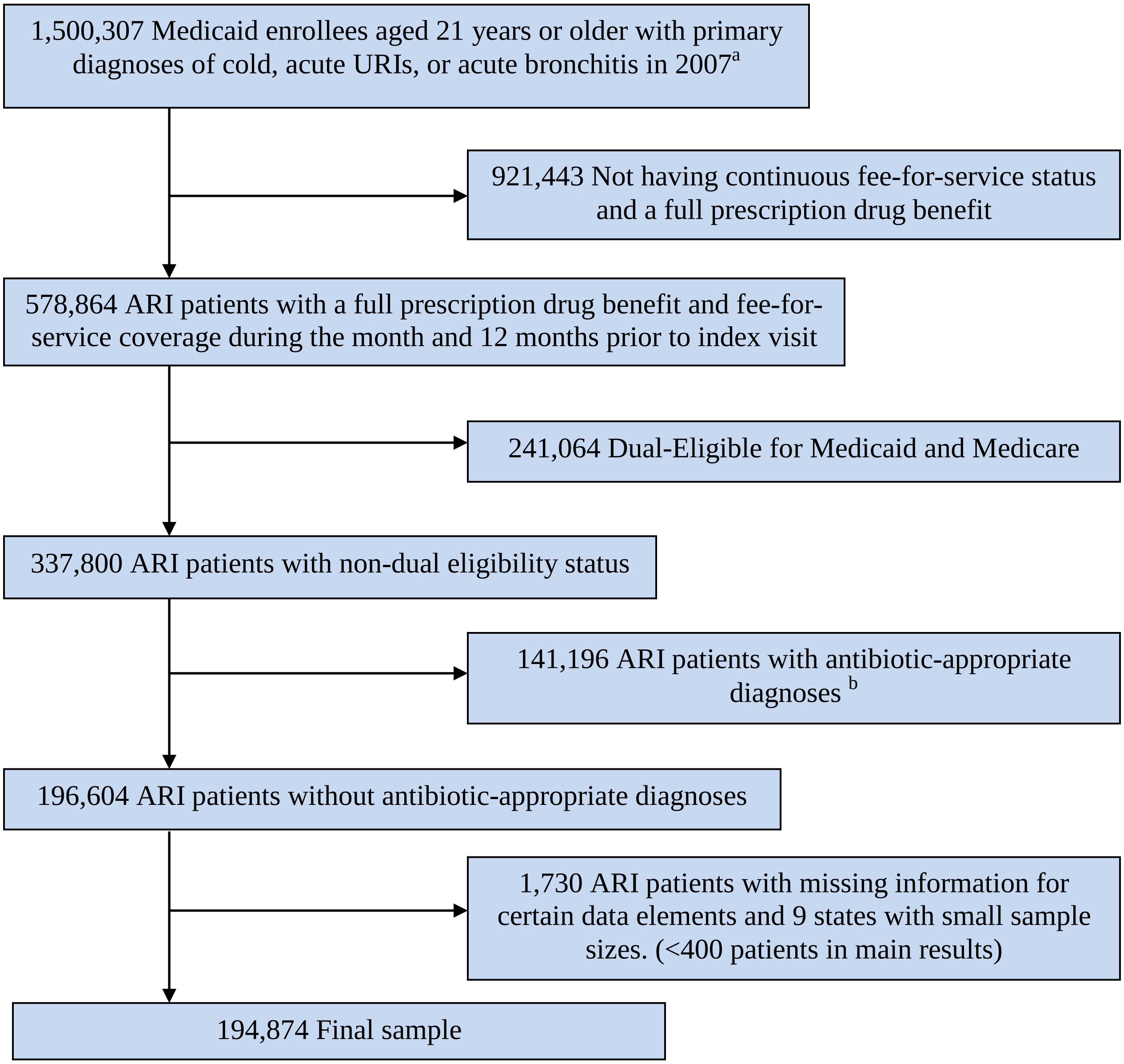
1,500,307 Medicaid enrollees aged 21 years or older with primary diagnoses of cold, acute URIs, or acute bronchitis in 2007[a]
921,443 Not having continuous fee-for-service status and a full prescription drug benefit
578,864 ARI patients with a full prescription drug benefit and fee-for-service coverage during the month and 12 months prior to index visit
241,064 Dual-Eligible for Medicaid and Medicare
337,800 ARI patients with non-dual eligibility status
141,196 ARI patients with antibiotic-appropriate diagnoses [b]
196,604 ARI patients without antibiotic-appropriate diagnoses
1,730 ARI patients with missing information for certain data elements and 9 states with small sample sizes. (<400 patients in main results)
194,874 Final sample

Technical Appendix Table. Sensitivity and Subgroup Analyses: Odds Ratios for PCP Density and CDC Get Smart Campaign

	Odds Ratio (95% Confidence Interval)						
	No. of PCP Physicians per 10,000 Persons[a]					Residence in State Participating in CDC Get Smart Campaign	
Variable	<2.2	2.2–3.4	3.5–4.7	4.8–6.5	>6.5	No	Yes
Main model	Referent	0.96 (0.87–1.07)	0.91 (0.80–1.04)	0.84 (0.73–0.96)	0.76 (0.66–0.88)	Referent	0.74 (0.62–0.88)
Varying on covariates							
Unadjusted odds ratio	Referent	0.98 (0.86–1.11)	0.95 (0.81–1.11)	0.81 (0.65–1.00)	0.71 (0.59–0.86)	Referent	0.75 (0.56–1.02)
Only control for patient characteristics[b]	Referent	0.99 (0.90–1.09)	0.92 (0.81–1.05)	0.82 (0.70–0.97)	0.79 (0.67–0.93)	Referent	0.76 (0.61–0.95)
Varying on dependent variable measure							
Link to the visit if antibiotic prescribed ≤3 days	Referent	0.96 (0.87–1.07)	0.91 (0.80–1.04)	0.83 (0.73–0.96)	0.76 (0.65–0.88)	Referent	0.73 (0.61–0.88)
Link to the visit if antibiotic prescribed ≤7 days	Referent	0.97 (0.87–1.08)	0.92 (0.81–1.04)	0.84 (0.74–0.97)	0.77 (0.67–0.89)	Referent	0.74 (0.62–0.89)
Subgroup analyses							
Age < 65 y	Referent	0.96 (0.87–1.07)	0.91 (0.80–1.03)	0.83 (0.72–0.95)	0.76 (0.65–0.88)	Referent	0.74 (0.61–0.89)
Without diabetes or congestive heart failure[c]	Referent	0.97 (0.87–1.09)	0.91 (0.80–1.03)	0.82 (0.72–0.94)	0.74 (0.64–0.86)	Referent	0.75 (0.62–0.90)

*Data are from the 2007 Medicaid Analytic Extract (MAX) files linked with the area resource file (ARF). Abbreviations: CDC, Centers for Disease Control and Prevention; CI, confidence interval.
[a]The OR is 0.98 (95% CI: 0.96-0.99; P-value< 0.001) if number of PCP Physicians per 10,000 Persons was coded as a continuous variable in the main model.
[b]Regressions were adjusted for age, sex, race, RxHCC, index diagnosis and quarter of index visit date.
[c]Visits with diagnoses of other comorbid conditions, such as diabetes (International Classification of Diseases, Ninth Revision, Clinical Modification [ICD-9-CM], codes 250, 357.2, 362.01, 362.02, 366.41) and congestive heart failure (ICD-9-CM code, 428.0) during the 1 year before the index visit date were also excluded.

References

1. Mainous AG III, Zoorob RJ, Hueston WJ. Current management of acute bronchitis in ambulatory care: The use of antibiotics and bronchodilators. Arch Fam Med. 1996;5:79–83. PubMed http://dx.doi.org/10.1001/archfami.5.2.79

2. Grijalva CG, Nuorti JP, Griffin MR. Antibiotic prescription rates for acute respiratory tract infections in US ambulatory settings. JAMA. 2009;302:758–66. PubMed http://dx.doi.org/10.1001/jama.2009.1163

3. Aspinall SL, Berlin JA, Zhang Y, Metlay JP. Facility-level variation in antibiotic prescriptions for veterans with upper respiratory infections. Clin Ther. 2005;27:258–62. PubMed http://dx.doi.org/10.1016/j.clinthera.2005.02.002

4. Gonzales R, Steiner JF, Sande MA. Antibiotic prescribing for adults with colds, upper respiratory tract infections, and bronchitis by ambulatory care physicians. JAMA. 1997;278:901. PubMed http://dx.doi.org/10.1001/jama.1997.03550110039033

5. Li J, De A, Ketchum K, Fagnan LJ, Haxby DG, Thomas A. Antimicrobial prescribing for upper respiratory infections and its effect on return visits. Fam Med. 2009;41:182–7. PubMed

6. McCaig LF, Hughes JM. Trends in antimicrobial drug prescribing among office-based physicians in the united states. JAMA. 1995;273:214. PubMed http://dx.doi.org/10.1001/jama.1995.03520270048030

7. Nyquist AC, Gonzales R, Steiner JF, Sande MA. Antibiotic prescribing for children with colds, upper respiratory tract infections, and bronchitis. JAMA. 1998;279:875. PubMed http://dx.doi.org/10.1001/jama.279.11.875

8. Zuckerman IH, Perencevich EN, Harris AD. Concurrent acute illness and comorbid conditions poorly predict antibiotic use in upper respiratory tract infections: A cross-sectional analysis. BMC Infect Dis. 2007;7:47. PubMed http://dx.doi.org/10.1186/1471-2334-7-47

9. Perz JF, Craig AS, Coffey CS, Jorgensen DM, Mitchel E, Hall S, et al. Changes in antibiotic prescribing for children after a community-wide campaign. JAMA. 2002;287:3103–9. PubMed http://dx.doi.org/10.1001/jama.287.23.3103

10. Mainous AG III, Hueston WJ. Davis MP PW. Trends in antimicrobial prescribing for bronchitis and upper respiratory infections among adults and children. Am J Public Health. 2003;93:1910–4. PubMed http://dx.doi.org/10.2105/AJPH.93.11.1910
